# Application of Photo-Electrochemically Generated Hydrogen with Fuel Cell Based Micro-Combined Heat and Power: A Dynamic System Modelling Study

**DOI:** 10.3390/molecules25010123

**Published:** 2019-12-28

**Authors:** Krisztian Ronaszegi, Eric S. Fraga, Jawwad Darr, Paul R. Shearing, Dan J. L. Brett

**Affiliations:** 1 Electrochemical Innovation Lab, Department of Chemical Engineering, University College London, London WC1E 7JE1, UK; krisztian.ronaszegi.10@ucl.ac.uk (K.R.); p.shearing@ucl.ac.uk (P.R.S.); 2 Centre for Process Systems Engineering, Department of Chemical Engineering, University College London, London WC1E 7JE2, UK; e.fraga@ucl.ac.uk; 3 Department of Chemistry, University College London, 20 Gordon Street, London WC1H 0AJ, UK; j.a.darr@ucl.ac.uk

**Keywords:** solar-to-hydrogen efficiency, photo-electrochemical cell, polymer electrolyte membrane fuel cell, micro-CHP, gas boiler, CO_2_ reduction

## Abstract

Photo-electrochemical (PEC) hydrogen generation is a promising technology and alternative to photovoltaic (PV)-electrolyser combined systems. Since there are no commercially available PEC cells and very limited field trials, a computer simulation was used to assess the efficacy of the approach for different domestic applications. Three mathematical models were used to obtain a view on how PEC generated hydrogen is able to cover demands for a representative dwelling. The analysed home was grid-connected and used a fuel cell based micro-CHP (micro-combined heat and power) system. Case studies were carried out that considered four different photo-electrode technologies to capture a range of current and possible future device efficiencies. The aim for this paper was to evaluate the system performance such as efficiency, fuel consumption and CO_2_ reduction capability. At the device unit level, the focus was on photo-electrode technological aspects, such as the effect of band-gap energy represented by different photo-materials on productivity of hydrogen and its uncertainty caused by the incident photon-to-current conversion efficiency (IPCE), which is highly electrode preparation specific. The presented dynamic model allows analysis of the performance of a renewable energy source integrated household with variable loads, which will aid system design and decision-making.

## 1. Introduction

The global increase in energy consumption can be partly alleviated by improving efficiencies of the incumbent technologies and through the use of alternative energy technologies. Future energy sources would ideally be inexhaustible, environmentally friendly and low cost. These are typically renewable energy sources such as wind, solar, geothermal energy, etc. Solar energy is one of the most promising renewable energy sources due to its abundance. However, intermittency is a major issue for solar energy, leading to the need to store the energy in electrical, chemical or thermal form. Storage in chemical form has the advantage of not suffering from loss during storage and can be easily transported. Effective storage is particularly important for applications where demand and supply need to be matched in different time scales. This includes the residential sector where significant energy saving and carbon dioxide reduction is required [1].

Hydrogen is a promising chemical energy storage vector that can be generated from renewable energy and water via a range of routes. There are four main methods for solar-to-hydrogen conversion: two direct and two indirect methods, which are determined by the number of sub-steps in the generation process or the number of units. Indirect methods include photo-biological and photovoltaic (PV)-electrolyser systems, while direct methods include photo-catalytic systems and photo-electrochemical (PEC) water splitting. Three out of four are feasible for use at a domestic scale, while the fourth one, which is the photo-biological method, is not considered a viable option [2,3]. PV-electrolyser systems have been demonstrated and proven feasible; however, such systems are yet to be commercialised. A disadvantage of this technology is that the system works indirectly, solar is converted to electricity and then the electricity used to generate hydrogen, which diminishes the overall efficiency.

Photo-catalysis and PEC systems are under active research and development; however, the technology is primarily at the laboratory scale with relatively low efficiencies demonstrated. The way in which the hydrogen is generated also varies between the different technologies; for example, photo-catalytic methods generate mixed gases, while PEC technology can be mixed or separated, a major advantage for the engineering of a practical device.

While a range of water splitting technologies continue to be developed, it is important to have a systematic means of comparing the methods in terms of efficiency, practicality, CO_2_ savings and techno-economic considerations. Assessing suitability for use in different applications and how they can best be combined and integrated into different systems is also paramount [4].

A complete system performance and economic analysis is required that considers the device within a system to achieve such a comparison. However, to the authors’ knowledge, there is no accepted mathematical model in the literature that fully describes the operation of ‘real’ photo-electrochemical devices in terms of generated photocurrent or solar-to-hydrogen efficiency, in the context of a dynamic simulation.

Solar water splitting involves complex processes, such as charge transfer kinetics at the semiconductor electrode-electrolyte interface, and is influenced by defects, dislocations and impurities in the photo-electrode [4]. In spite of these complexities, attempts have been made to describe performance in terms of ideal analytical equations. Models of PEC performance can be divided into those that primarily consider techno-economic issues and those that are focused on the scientific development of the technology.

Models for techno-economic analysis are typically based on rudimentary formulas, often using ‘black-box’ models to describe the efficiency for photo-electrochemical devices. These are often composed of hypothetical constants, thermodynamic efficiency limit or some measured efficiencies [5,6]. Since this kind of approximation is not a function of any parameters, such studies are mainly concentrated on static cost or techno-economic calculations that consider the value of the hydrogen generation, but not its subsequent consumption as part of a broader system [7,8]. Examples include Wingens et al. [9] who introduce some dynamic components in the comparison of PEC and PV units, with other studies comparing the cost-effectiveness of PEC technology with PV-electrolyser combinations, but without a detailed modelling framework [6].

The second main group of models focus on scientific development of PEC technology to explore parameter dependency and describe mechanistic effects. However, these models are not typically designed for techno-economic or whole-system calculations. In general, they contain parameters that are difficult to determine or highly dependent on the material set employed in the device. These scientific models can be separated further into sub-groups according to approximations of problems. Some models are based on polarisation curve generation [10,11,12,13]. Within these models, some put the emphasis on the semiconductor [12] while others on the charge transfer at the interface [11]. Papers which are focusing on the semiconductor term, in general, use fitted data for the overpotential at the charge transfer at the interface which is specific for those materials or they use Butler–Volmer kinetic behaviour to determine the overpotential. Other sub-group models consider thermodynamic limitations derived from the detailed balance model made by Shockley and Queisser for a p–n junction solar cell [14]. This model has been expanded by many authors, with Ross and co-workers converting the model into a general form that can describe a range of photo-electrochemical systems [15,16].

The present work assesses the performance and the viability of a single band-gap photosensitive semiconductor PEC device as part of a domestic micro-CHP (micro combined heat and power) system. A modified version of the Ross–Hsiao model is used to describe PEC performance and the time-varying solar resource and heat and electricity load profiles of a domestic residence are considered. In this way, the study represents the first dynamic analysis of a PEC device within a micro-CHP system using a relatively sophisticated model of PEC performance.

It should be noted that the polymer electrolyte fuel cell operation is modelled based on conventional materials and provides representative performance estimates, alternative materials and operational strategies can deliver higher efficiency/performance. For example, the use of polymer-ceramic composites [17,18].

## 2. Modelling Methodology

### 2.1. System Description

**Figure 1:**
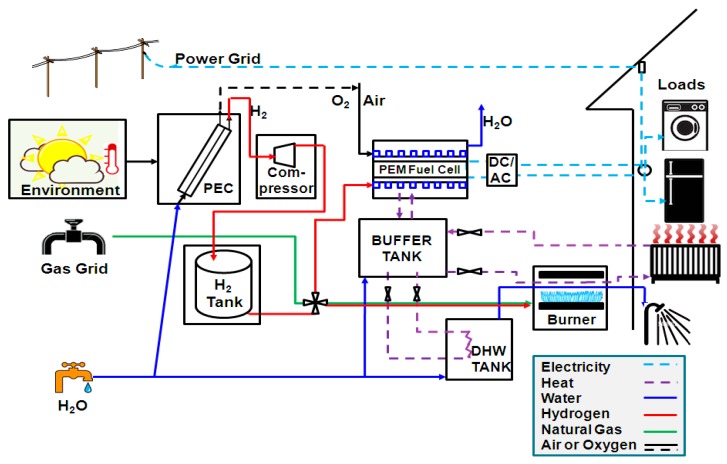
Schematic of a potential solar-photo-electrochemical (PEC)-hydrogen domestic micro-CHP (micro-combined heat and power) system.

A representative photo-electrochemical solar-to-hydrogen system for energy supply to a domestic application is shown in Figure 1; this forms the basis of the model presented below. The system comprises a photo-electrochemical unit (or array); gas auxiliary units such as a compressor and a high pressure storage tank for hydrogen; a polymer electrolyte membrane fuel cell (PEMFC) with an inverter; a thermal buffer/storage tank; a domestic hot water (DHW) tank; a gas boiler assumed to be able to operate on both hydrogen and natural gas; and grid supplies of electricity, water and natural gas. A storage tank for oxygen is optional, depending on the cost-efficiency balance for the extra gas tank vs. improved efficiency of the fuel cell.

Fuel cells offer an excellent option for micro combined heat and power (CHP) for residential applications [19,20]. Fuel cell micro-CHP is particularly suitable for low heat-to-power ratio applications such as during summer months and in well-insulated homes. A PEM fuel cell was chosen because of its good dynamic responsiveness to load variation and on/off cycling (compared with the intermediate temperature solid oxide fuel cells, the other main fuel cell technology being developed for micro-CHP applications [21]).

The model requires that the energy demands (electricity, hot water and space heating) for a UK household are met using the solar resource available, with recourse to grid supplies, as necessary. In the case when there is surplus hydrogen, then it is stored. If there is not enough hydrogen in the system to supply demands, then the system first covers electricity demand from the grid and the generated heat is stored in a buffer tank. The system does not store electricity; only heat and hydrogen are stored. This means that the CHP system is electricity demand-driven. After electricity demand has been serviced, if there is a deficit in heat available from hydrogen, both hot water and space heating is supplied via natural gas from the grid, using a boiler which is assumed to work on both fuels with the same efficiency.

### 2.2. System Model

The overall system model is composed of individual device models for each unit in the system. Established models and parameter values were taken from the literature or mass and energy balances were derived when existing models were not available. Environmental parameters, such as solar radiation and ambient temperature, as well as load profiles were used as time-varying inputs with hourly resolution.

#### 2.2.1. Photo-Electrochemical Model

Three different photo-electrochemical models were considered: (1) Ross–Hsiao model, (2) Modified Ross–Hsiao model, and (3) the so-called ‘constant efficiency’ model.

The Ross–Hsiao model [16,22,23] is a thermodynamic efficiency model for photo-electrochemical systems where the model shows the thermodynamic limits for solar conversion to chemical energy. The power for such a solar converter system is equal to the quantity of photoproduct multiplied by the free-energy difference between the initial state of the material and the product state. This model assumes that there are three states in the system: a ground state (initial state); an excited state, which is only radiatively coupled to the ground state and a product state, which is kinetically coupled to the excited state by a chemical reaction path. According to the model, the maximum power can be obtained when the rate of light emission is equal to the rate of light absorption between electronic states. The rate of conversion of solar radiation to chemical energy is given by Equation (1),

(1) $$P=J_{S}\;\times \;\mu \;\times \;\left(1-\varphi_{loss}\right),$$

where *P* (W m^−2^) is the total power converted to chemical energy, *J_S_* (number of photons m^−2^ s^−1^) is the flux of absorbed photons, *μ* (J (number of photons)^−1^) is the chemical potential generated in the system. In the last term, *Φ_loss_* is the quantum yield that is lost via radiative mechanism from the excited state, but without leading to a photoproduct. The 1-*Φ_loss_* term thus represents the fraction of the absorbed photons which turn to photoproduct. In the Ross–Hsiao model, it was assumed that there is no non-radiative loss such as electron–hole recombination in the bulk or in surface states. This means the internal conversion, i.e., incident-photon-to-current efficiency (*η*_IPCE_) or briefly quantum efficiency, was assumed to be 100%.

The thermodynamic efficiency is given by Equation (2), where the denominator describes the total solar energy over all wavelengths which entered the PEC system.

(2) $$\eta_{TD}=\frac{P}{\int_{0}^{\infty }\varphi \left(\lambda \right)d\lambda },$$

**Figure 2:**
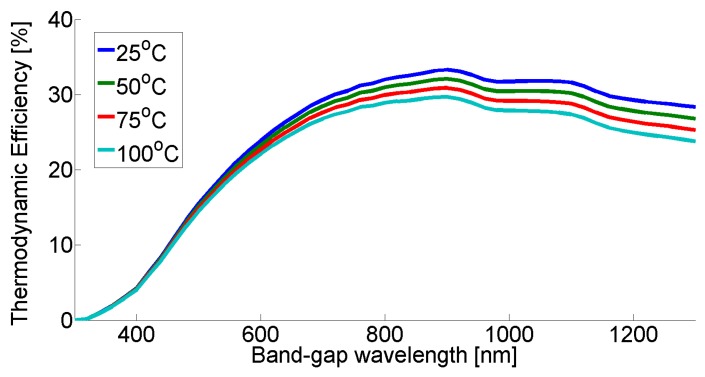
Efficiency curves of the Ross–Hsiao model as a function of temperature.

In Figure 2, thermodynamic efficiency profiles can be seen according to the Ross–Hsiao model for different PEC cell temperatures.

The hydrogen generation rate can be calculated by using the heat of combustion of hydrogen, $\Delta H_{C}^{o}$ (Wh kg^−1^), (Equation (3)). *A_PEC_* (m^2^) is the area of the cell and *G* (W m^−2^) is the solar radiation intensity.

(3) $$m_{H2}=\frac{A_{PEC}\eta_{TD}G}{\Delta H_{C}^{o}},$$

The solar-to-hydrogen efficiency (in Equation (3)) represents an ideal limit in the conversion; however, there are loss factors which need to be taken into consideration to obtain a more realistic model; this will be presented as the ‘modified Ross–Hsiao model’ in the present paper. The loss factors are now described.

Since the Ross–Hsiao model does not describe the PEC unit as a device, the first loss comes from the optical loss which is mainly due to reflectance of light from the optical window and the electrode surface. Solar collectors have data measured for this kind of light fraction loss, represented as the optical efficiency (*η*_0_) [24]. This approximation will be used in the modified Ross–Hsiao model.

Inside the photo-electrode, if there is non-radiative loss, such as electron–hole recombination, then in contrast to the Ross–Hsiao model the incident-photon-to-current efficiency (IPCE or also *η*_IPCE_) is not 100%. The IPCE varies depending on the chemistry and material processing, and is a function of wavelength. To be considered in Equation (1), *J_S_* is defined according to Equation (4),

(4) $$J_{S}=\int_{0}^{\lambda g}\eta_{IPCE}\left(\lambda \right)N_{S}\left(\lambda \right)d\lambda ,$$

where the new *J_S_* (number of photons m^−2^ s^−1^) is the flux of absorbed photons corrected by the quantum efficiency, *N_s_* (number of photons m^−2^ s^−1^ nm^−1^) is the incident solar photon flux and *λ_g_* (nm) is the band-gap wavelength of the semiconductor.

*η_IPCE_* can be based on measured data or on calculations. For calculated data, a theoretical approximation can be used, such as the model presented by Ghosh and Maruska [23]. In the modified Ross–Hsiao model for non-radiative loss, the Ghosh and Maruska model is used according to Equation (5) [25,26].

(5) $$\eta_{IPCE}=\eta_{QE}\left(\left[1-exp\left(-kl_{b}\right)\right]+\frac{k}{k+\beta }exp\left(\beta l_{b}\right)\times \left(exp\left[-\left(k+\beta \right)\right]l_{b}-exp\left[-\left(k+\beta \right)\right]h\right)\right)$$

where *η_QE_* is the quantum conversion efficiency, *k* (cm^−1^) is the absorption coefficient, *l_b_* (cm) is the width of space charge region, *β* (cm^−1^) is the inverse of the particles’ diffusion length and *h* (cm) is the semiconductor layer thickness. The first term in the main bracket (1-*exp*(-*kl_b_*)) is the fraction of the generated carriers within the barrier region and the second term in the main bracket (the rest) is the fraction of carriers generated in the bulk region. Parameters change according to the preparation method and can be obtained from measured data with regression analysis.

**Figure 3:**
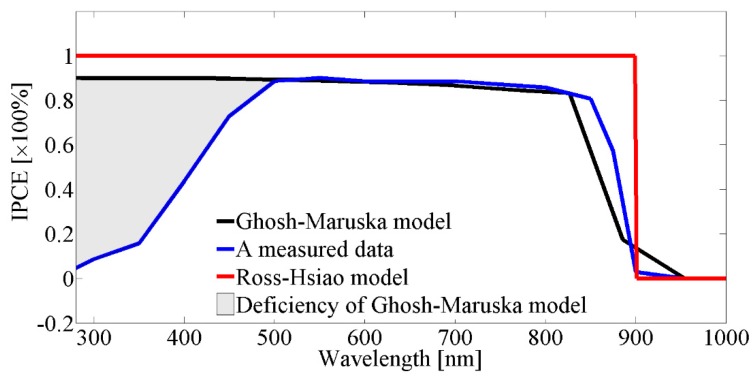
Incident photon-to-current conversion efficiency (IPCE) curves according to models and measured data at GaAs photo-electrode.

IPCE curves can be seen in Figure 3, based on the Ross–Hsiao model and the Ghosh–Maruska model, together with measured data for the case of a GaAs semiconductor.

Figure 3 shows that with the Ross–Hsiao model the IPCE was uniform and 100% due to the lack of non-radiative loss. A real IPCE curve can be seen for GaAs [27]. For most semiconductors, high-energy photons have low or near-zero quantum efficiencies. The Ghosh–Maruska model is not able to describe that part of the IPCE spectrum (shaded part on the graph). This is a systematic deficiency of the model where the extent of it changes from material to material.

The last loss which has been considered relates to the kinetic loss of the charge transfer (*η_chem_*) at surface reactions. For this loss term, the model used was from Bolton [23]. This model assumes that 0.8 eV must be lost in each photochemical step. The model equation can be seen in Equation (6).

(6) $$\eta_{chem}=\frac{\Delta G}{\Delta G+0.8neN_{0}}$$

where Δ*G* is the free-energy change in the overall reaction (Δ*G* = 237 kJ mol^−1^ at the water-splitting reaction), *n* is the number of electrons transferred (*n* = 2 for water splitting), *e* (C) is the elementary charge and *N_0_* (mol^−1^) is Avogadro’s constant.

The overall efficiency for the modified Ross–Hsiao model is the same as the solar-to-hydrogen efficiency (STH) for the PEC cell. The applied STH efficiency used here is given by Equation (7).

(7) $$\eta_{STH}=\eta_{TD}\times \eta_{IPCE}\times \eta_{0}\times \eta_{chem},$$

The overall efficiency in Equation (7) can be extended with additional terms to get a more accurate model. Such terms could come from geometry aspects of the cell design, optical aspects caused by bubble formation of generated gases and age effects of photo-electrodes caused by photo- and electrochemical corrosions. These terms are not considered in this work because of the lack of intensive research and available PEC cell unit.

The third model is the ‘constant efficiency’ model. This considers the efficiency to be a constant number, which comes from the yearly average of the PEC thermodynamic efficiency. This constant efficiency does not change with cell temperature and is not affected by the changes in the solar spectrum, which arise from weather/environmental effects. In Figure 2, we can see that the temperature has a higher effect on higher band-gap wavelength materials, thus the ‘constant efficiency’ model can show the extent of this at different band-gap wavelength materials. The photo-electrochemical cell operated at ambient temperature without any heat accumulation.

#### 2.2.2. Hydrogen Gas Tank Model

The hydrogen gas tank was modelled by a simple mass balance and the Van der Waals equation, according to certain assumptions: (i) the tank operates when the pressure inside is higher than atmospheric pressure. This means that there may be hydrogen generation taking place but unless the tank is higher than atmospheric pressure no hydrogen can be delivered to the fuel cell or burner. (ii) The tank operates under ambient temperature. (iii) The pressure is limited to 200 bar; venting occurs if in excess of this value.

#### 2.2.3. PEM Fuel Cell Model

For the PEM fuel cell, a lumped model was chosen with constant operation temperature (*T_cell_* = 80 °C) and atmospheric pressure. Inlet gas composition was assumed to be 90% hydrogen and 10% water vapour in the anode side and 21% oxygen and 79% nitrogen in the cathode side.

The model generates a polarisation curve by adopting equations and the parameters reported in Spiegel [28]. The main formulas are shown in Equations (8)–(12).

(8) $$V_{cell}=E_{Nernst}-\eta_{act}-\eta_{ohmic}-\eta_{conc},$$

And

(9) $$\eta_{act}=\sum \frac{RT_{cell}}{z_{i}\alpha_{i}F}log\left(\frac{i}{i_{0,i}}\right)$$

(10) $$\eta_{ohmic}=iR_{in}$$

(11) $$\eta_{conc}=\frac{RT_{cell}}{z_{i}F}ln\left(\frac{i_{L}}{i_{L}-i}\right)$$

(12) $$E_{Nernst}=1.229-0.846\times 10^{-3}\left(T_{cell}-298.15\right)+\frac{RT_{cell}}{z_{a}F}log\left(\frac{p_{H2}p_{O2}^{1/2}}{p_{H2O}}\right)$$

where the cell voltage (*V_cell_* [V]) based on the difference between the Nernstian potential (*E_Nernst_* [V]) and the various overpotentials, such as activation overpotential (*η_act_* [V]), ohmic overpotential (*η_ohmic_* [V]) and the concentration overpotential (*η_conc_* [V]). The further parameters are the Regnault constant (gas constant, *R* [J mol^−1^ K^−1^]), cell operation temperature (*T_cell_* [°C]), the number of electrons involved in the anode and cathode reaction (*z_i_*={*z_a_*,*z_c_*}), the transfer coefficient for the reaction of interest (αi), the Faraday constant (*F* [C mol^−1^]), the current density (*i* [A cm^−2^]), the exchange current density (*i_0_*_,i_ [A cm^−2^]) for both the hydrogen oxidation reaction (HOR) and oxygen reduction reaction (ORR), the internal resistance (*R_in_* [Ω cm^−2^]), the limiting current density (*i_L_* [A cm^−2^]) and the partial pressure for the components (*p_i_* [Pa]).

The characteristic polarisation curve of the cell provides the specific power (*P_cell_* [W cm^−2^]) from Equation (13).

(13) $$P_{cell}=V_{cell}\times i,$$

The next step is to determine the fuel cell electrode area for the demand/hydrogen supplied. It can be calculated from the cell specific power and the maximum power (*P_max_* [W]) of the fuel cell, which is the highest output that can support electricity demand. *P_max_* depends on the electricity demand of the family. Since any electrochemical cell produces DC current and voltage, this current must be converted to AC to be used in a household for equipment which is normally powered from the grid. An inverter does this with high conversion efficiency, *η_inv_*. Equation (14) tells us the total area (*A_tot_* [cm^2^]) which needs for the calculation of hydrogen quantity to cover the actual electricity demand.

(14) $$A_{tot}=\frac{P_{max}}{P_{cell}\eta_{inv}},$$

Whether the total area is concentrated into one single cell or in a stack with many smaller area individual cells linked together in series is irrelevant in terms of energy calculation in the lumped model.

In Equation (14), the specific power (*P_cell_*) can be different in time according to the operation mode of the fuel cell. However, it does not mean that the total area of the fuel cell changes during the yearly simulation since the cell is a fixed geometrical unit. The fuel cell can operate with constant output voltage and so the specific power is fixed during operation or the voltage can be changed, thus the output specific power will change as well. At constant voltage mode, an optimal voltage value is selected which is also referred to as the nominal voltage or operation voltage (*V_op_* [V]). The fuel cell model continuously checks the hydrogen level in the tank and if it is insufficient to deliver the power required, the grid is used to make up the shortfall. During simulations, these two operation modes are also called full and partial power modes.

Calculating the amount of hydrogen (see Equation (15)) from the required power, the current is determined by the fuel cell power density-current density curve. From the current, the required hydrogen quantity can be determined using Faraday’s law (see Equation (15)) assuming 100% utilization factor.

(15) $${\dot{n}}_{H2}=\frac{iA_{tot}}{z_{a}F},$$

During the operation of the fuel cell, heat is generated which can be used to meet thermal demands by utilizing it in the CHP system. Equations (16) and (17) give the generated heat flow (*Q_cell_* [W]) [20] at full power and partial power mode, respectively. For the partial power mode, the generated heat is proportional to the actual power (*P_actual_*) for which the hydrogen is just enough to cover and not the maximum power.

(16) $${\dot{Q}}_{cell}=\dot{P}{\left(\frac{E_{rev}}{V_{op}}-1\right)}_{max},$$

(17) $${\dot{Q}}_{cell}={\dot{P}}_{actual}\left(\frac{E_{rev}}{V_{actual}}-1\right),$$

#### 2.2.4. CHP Operation with Thermal Units

Thermal units include a buffer tank and a hot water tank, as shown in Figure 1. The operation of the CHP system is such that the heat from the fuel cell is accumulated in the buffer tank as a central collector unit. The heat from the buffer tank can be used for space heating or hot water supply, in which case the heat is transferred to a hot water tank via an internal heat exchanger such that the liquid medium in the closed buffer tank system is not mixed with the water in the hot water tank. This kind of integrated buffer tank system has a positive effect on the cogeneration system performance, as reported by Beyer and Kelly [29].

The water inlet temperature to the system from the mains supply is taken to be 10 °C (*T_MAIN_*) and assumed to be independent of seasonal effects. The hot water outlet temperature was chosen to be 60 °C at the consumer side. This value is enough for setting the required temperature for given applications which typically vary between 35 °C and 60 °C. A value of 60 °C also mitigates against legionella bacteria [30].

The thermal system model is based on heat balance that incorporates heat accumulation in the two thermal storage units (see Equations (24) and (25)). The driving force is the temperature difference between units. Heat transfer between units was modelled by using simplified counter-flow heat exchange with uniform wall temperature (see Equations (18), (20) and (22)). The total heat transfer is proportional to the inlet and outlet temperature differences in the heat exchanger pipes (see Equations (19), (21) and (23)). The only heat source in the CHP storage system is the fuel cell heat, which exits at 80 °C.

Buffer tank-Radiators heat transfer:

(18) $$T_{Buff,in,SH}=\left(T_{Buff,out}-T_{SET}\right)exp\left(-\frac{U_{rad}A_{rad}}{{\dot{m}}_{SH}c_{p,H2O}}\right),$$

(19) $${\dot{Q}}_{SH}={\dot{m}}_{SH}c_{p,H2O}\left(T_{Buff,out}-T_{Buff,in,SH}\right),$$

Buffer tank-Hot Water tank heat transfer:

(20) $$T_{Buff,in,HW}=\left(T_{Buff,out}-T_{HW}\right)exp\left(-\frac{U_{HE}A_{HE}}{{\dot{m}}_{HW}c_{p,H2O}}\right)+T_{HW},$$

(21) $${\dot{Q}}_{HW}={\dot{m}}_{HW}c_{p,H2O}\left(T_{Buff,out}-T_{Buff,in,HW}\right),$$

Fuel cell-Buffer tank heat transfer:

(22) $$T_{FC,in}=\left(T_{FC,out}-T_{Buff}\right)exp\left(-\frac{U_{HE}A_{HE,FC}}{{\dot{m}}_{FC}c_{p,H2O}}\right)+T_{Buff},$$

(23) $${\dot{Q}}_{FC}={\dot{m}}_{FC}c_{p,H2O}\left(T_{FC,out}-T_{FC,in}\right),$$

Heat balance for the buffer tank (Buff) and for the hot water tank (HW):

(24) $$V_{Buff}\rho_{H2O}c_{p,H2O}\frac{dT_{Buff}}{dt}={\dot{Q}}_{FC}-{\dot{Q}}_{SH}-{\dot{Q}}_{HW},$$

(25) $$V_{HW}\rho_{H2O}c_{p,H2O}\frac{dT_{HW}}{dt}={\dot{Q}}_{HW}-{\dot{v}}_{LOAD}\rho_{H2O}c_{p,H2O}\left(T_{HW}-T_{MAIN}\right),$$

where *T_SET_* (°C) is the required room temperature. When there is no hourly load or the temperature in the buffer tank is less than TSET, then the flowrate (*ṁ_SH_*) is zero in the buffer tank and radiators loop. A single total radiator area (*A_rad_*) is considered. *U_rad_* (W m^−2^ K^−1^) is the overall heat transfer coefficient of the radiators and UHE is the overall heat transfer coefficient of the heat exchangers in the tanks. *V_HW_* (m^3^) is the volume of the hot water tank and *V_Buff_* the buffer tank. ${\dot{v}}_{load}$ (m^3^ s^−1^) is the flow rate of hot water in a given hour, *ρ* (kg m^−3^) is the density of water, *c_p,H2O_* (J kg^−1^ K^−1^) is the specific heat capacity of water and *t* (s) is time.

The multi-fuel burner provides additional heat and is taken to have an efficiency of 90%. The boiler model checks the hydrogen content in the gas tank and switches to natural gas when the hydrogen is consumed. The quantity of fuel required is calculated using Equation (26), where *Q_deficit_* (J) is the deficit energy that is needed for the remaining load after reducing the energy from the buffer tank or the hot water tank. For the hot water tank, the deficit is the temperature difference between the actual temperature in the tank and 60 °C, as the final temperature requirement. *HHV_H2/NG_* (J kg^−1^) is the higher heating value of the given fuel gas and *η_boiler_* is the boiler efficiency. It was assumed that the flue gas temperature is the same as the fuel temperature, i.e., a condensing boiler was used.

(26) $$m_{H2/NG}=\frac{Q_{deficit}}{HHV_{H2/NG}\eta_{boiler}},$$

### 2.3. Input Data

#### 2.3.1. Environmental Parameters

The calculations of PEC and system efficiency require characterisation of the solar resource and ambient temperature at different times of day and year for a given global location. The ASHRAE Clear-Sky model was used to estimate the solar resource by considering the geometrical orbital of the Earth during yearly periods [31,32].

The Clear-Sky model can only predict solar intensity at a given time and location; it does not describe weather effects (i.e., transient cloud cover). Weather is chaotic and hard to predict; here a combination of real data and stochastic methods are used to simulate the weather effect. The Markov Transition Matrix technique is invoked to do this [33]. The technique uses the clearness index which comes from measured data over a given time period. The information is stored in matrix form where the elements of the matrix represent the probability of a cloudiness transition event between any two days.

**Figure 4:**
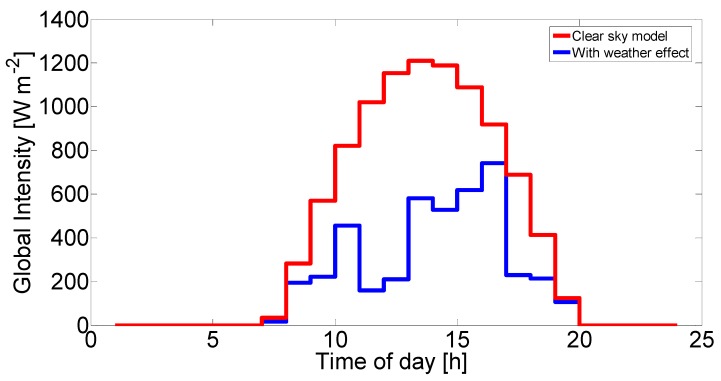
Global solar radiation in hour resolution with the weather effect generated using the MTM technique and the envelop curves without the weather effect.

Open source code was used for the clearness index [34]. In Figure 4, the generated solar radiation can be seen according to the code in minute resolution, which was then averaged to obtain the hourly resolution required for our simulation. Parameters were set for the given solar data: London, 14 July and global solar intensity.

In addition to the effect of weather, the incident solar spectrum must be defined. The solar spectrum needs the PEC efficiency calculation from which the *J_S_* (flux of absorbed photons) are derived. The SMARTS open-access program was used to generate spectra for given location, time and varied conditions [35]. Simulations of solar spectra were generated at sea level in the UK with different air mass (AM) values (AM: 1, 1.5, 2, 3 and 5, which represent different times of day). For atmospheric pollution, only one value was used over the full year, which was the default setting of the software for an urban area.

Ambient temperatures were taken using measured 5-min-data from the UK Carbon Trust CHP Accelerator project [36].

#### 2.3.2. Load Profiles

**Figure 5:**
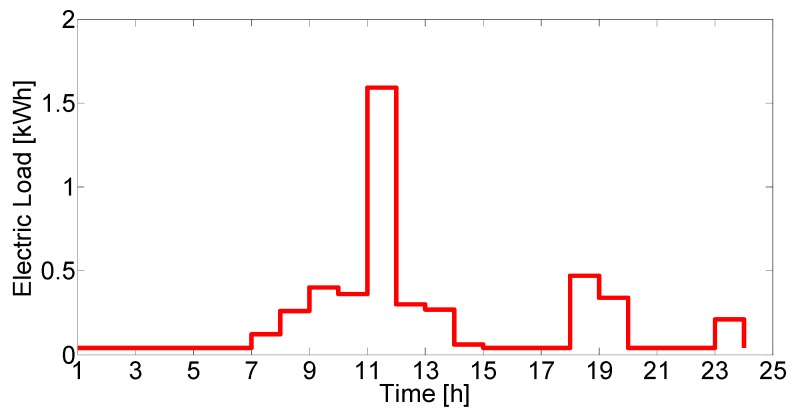
Electric load on a typical day during the year.

The loads consist of electricity, hot water and space heating, with typical UK consumption behaviour. The electricity load (Figure 5.) was simulated using an open-source program from Loughborough University [34]. The program operation is based on the method described by Yao and Steemers for domestic energy load profile generation [30].

Simulating hot water demand profiles is more challenging than electricity profile generation. The residents’ behaviour is the most significant factor in hot water usage. For total demand approximation, a rule of thumb method can be used. In recent work, 68 litres of hot water per person per day was used [37]. For specific profiles, occupancy patterns were generated considering a full worker household where it was assumed that consumption can occur mainly in the morning (7:00–9:00) and evening (17:00–23:00) periods [38]. Within the occupancy periods, a Monte Carlo method was used to represent the uncertainty of actual hot water consumption.

**Figure 6:**
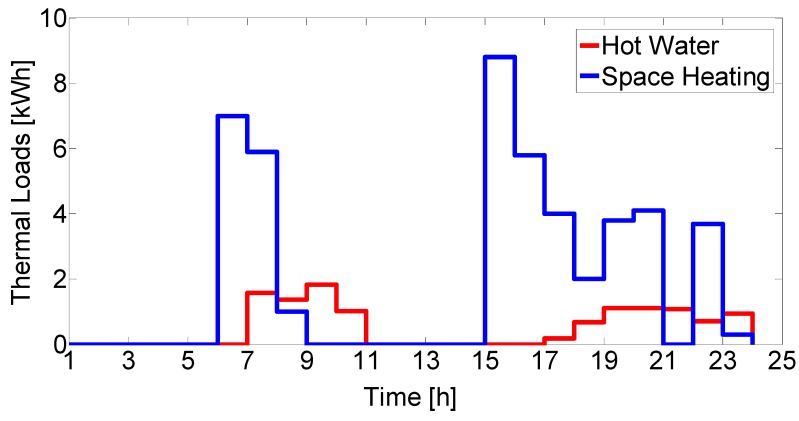
Typical daily thermal loads (winter). The demand for space heating changes by following the degree-hours during the year.

Space heating energy requirements were estimated by using a degree-hours method. The model is based on the difference between the internal setpoint temperature in the house which is a function of human comfort level and ambient temperature (*T_amb_*) at the given location [39,40]. This is the temperature difference which keeps the balance between the environment and the house needs. Figure 6 shows the calculated heat requirement for a winter day.

### 2.4. Simulation Data

The previous sub-sections include the mathematical models of components of a PEC system together with the auxiliary units which comprise a total energy system for a dwelling. The models were implemented in MATLAB. Each component of the energy system is able to run separately to allow for individual unit analysis but they can all also be combined into a single complex model for the analysis of the whole system.

#### Case study Information

A photo-electrochemically generated hydrogen system was used to meet some of the energy demands for a household with three occupants, as can be seen in Figure 1. This household was assumed to be in London, UK, where there are grid, water and natural gas mains supplies. The parameters and settings used for solar data and load profiles generation, as well as the building performance, are given in Table 1. The size of the house was randomly chosen for three people while coefficients were used from Mehleri et al. to the total heat loss coefficient [39].

The photo-electrochemical water-splitting panel was assumed to be fix mounted on the roof with the same tilt angle orientation as the latitude to obtain the maximum solar energy accumulation during the whole year. A typical available roof area in London is ~15–20 m^2^; here 18 m^2^ was used for PEC unit size [41].

Further parameters and settings for units in the model library are given in Table 2, Table 3 and Table 4. Parameters for the PEC unit and its auxiliary units are given in Table 2.

Simulations were carried out using four PEC electrode materials to represent the range of existing and target efficiencies for PEC devices. TiO_2_ is regarded as a conventional material with CdS, GaInP_2_ and GaAs representing target efficiencies for hydrogen production in future devices. IPCE data for the simulations were obtained from the literature [26,27,42,43].

It was assumed that the pressure in the hydrogen tank is not higher than 200 bars for safety reasons and that the hydrogen tank size of 2 m^3^ was chosen based on commercially available products.

CHP group device parameters are given in Table 3 with some selected data from the literature [29,30].

It was assumed that the maximum energy content for the buffer tank is the amount of heat which could be stored when the water content is heated to the maximum temperature. It was also assumed that the boiler can work with both hydrogen and natural gas and that the efficiency is the same for both.

Parameters related to electricity generation are given in Table 4, with some selected data from the literature [27,28,37].

With the given parameters, the PEM fuel cell overall electrode area is 1571 cm^2^. The following greenhouse gas (GHG) emission factors were used for electricity generation (0.5246 CO_2_ kg kWh^−1^) and heat (0.1836 CO_2_ kg kWh^−1^) [44].

## 3. Results

This paper deals with how photo-electrochemically generated hydrogen is able to cover loads of a three-person household. Dynamic simulations were carried out by using one-hour time steps during a full year. The selected model and its modification give a realistic picture about hydrogen productivity of the technology. Different band-gap energy electrode materials were analysed to obtain a picture of where the given technology is now and needs to progress to in the future. Results also show the effect on reducing fossil-based primary fuel and CO_2_ emissions.

### 3.1. Model Analysis

**Figure 7:**
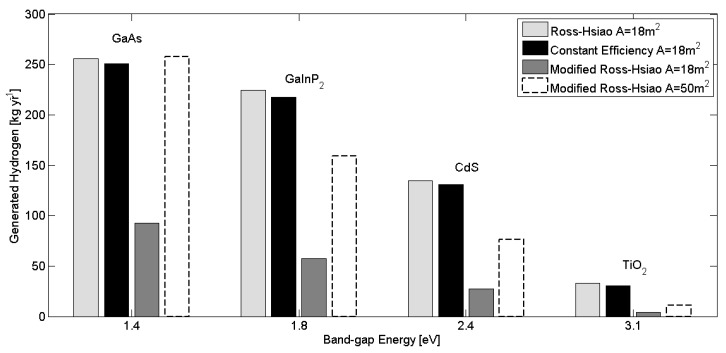
Annual generated hydrogen can be seen as a function of band-gap energies in the cases of analysed model approximation.

The hydrogen generation capacity of the photo-electrochemical unit can be seen in Figure 7.

The four bars represent the different PEC models as a function of band-gap energies. Four band-gap energies were analysed: TiO_2_ [45], CdS [25], GaInP_2_ [21], GaAs [46]. These will not necessarily be used for water splitting in technological devices because of their photo instability in the electrolyte, but it is useful to have an indication of the possibilities for hydrogen generation if they were. The most generated hydrogen quantities (bright grey bars) come from the ideal Ross–Hsiao model when there is only radiative loss. In the case of TiO_2_, which represents the current available capacity of this technology, the generated hydrogen is about 33 kg per year, while it is about 256 kg for GaAs, which could be used as a future target. The second largest hydrogen generation comes from the other idealised model approximation, which is the constant efficiency model that uses the Ross–Hsiao approach, with annual average efficiency used for each band-gap material. The differences between these two approximations are that in the efficiency model there are no temperature effect and no change in the solar spectrum due to pollutants and the natural annual climate change. Results for these two approximations show the same order of magnitude in hydrogen quantity. However, the simplified constant efficiency model shows a systematic underestimation relative to the more complex (Ross–Hsiao) model. The extent of underestimation changes by the band-gap energies, decreasing with decreasing band-gap energy.

The smallest (dark grey) bars show the modified Ross–Hsiao model, where further losses were taken into account. The neglected terms lead to a significant reduction in the quantity of hydrogen produced. With the more realistic model, the generated hydrogen is ~4 kg for TiO_2_ instead of 33 kg and it is about 93 kg for GaAs instead of 256 kg. These values are consistent with experimental results, thus this model approximation is accepted to be sufficiently accurate.

Bars with a dash line show how much hydrogen can be generated if a larger solar collection area were to be used. In the case of 50 m^2^, a TiO_2_-based device could generate about 11 kg, while for GaAs the larger area puts the production rate on a par with predictions from the ideal model.

By using the modified model in the next subsection, one can see how the PEC unit participates in an integrated domestic CHP system.

### 3.2. Application of the Modified PEC Model

The primary operation strategy was characterized as an ‘electric following’ or ‘electric-driven’ operation strategy. The thermal load is serviced by heat recovery from the CHP, supported by direct gas supply from the grid.

The two fuel cell modes were: full power mode, which only works when there is enough hydrogen, but then it gives maximum power and heat. The partial power mode, where the fuel cell works with the available hydrogen and the remaining power required, is supplied by the grid. For these measurements, the most efficient photo-electrode (GaAs) was chosen with complete electricity (E) and thermal demands (H, SH). Results are summarised in Table 5.

At 18 m^2^ PEC size, the required grid for full and partial power mode usage are 2492 kWh (F) and 1658 kWh (P), respectively. According to these results, the partial power operation mode has better benefits in terms of grid quantity and CO_2_ emissions. There is no cost optimisation considered in this paper.

The maximum temperatures are different during the year for the two operation modes, but the mean values are the same. This is a consequence of low operation hours of the fuel cell if insufficient hydrogen is generated, even with the more efficient photo-electrode.

In the case when the PEC size is 50 m^2^, the grid usage is also less at partial power mode, although more hydrogen was used for electricity generation at full power mode. This is because when the fuel cell operates it uses a fixed amount of hydrogen to provide the maximum power, even though the actual required power is less. With this PEC size, the CO_2_ emission is less still less at partial power mode, although the difference is not less at ~2%.

In further analysis, electrode efficiencies, demand variation and utilization of CHP mode were investigated. Table 6 and Table 7 show the results of the yearly simulations with 18 m^2^ PEC size, 50 litre buffer tank size, partial power fuel cell mode.

It can be seen that for TiO_2_, only 4 kg hydrogen can be generated during a year. According to the more realistic modified Ross–Hsiao model, the efficiency is about 0.45%, resulting in the low hydrogen production for an 18 m^2^ electrode area and reducing the grid requirement from 2540 kWh to 2427 kWh. This corresponds to a CO_2_ emission decreases from 1331 kg to 1273 kg. Since the hydrogen generated is so small, the generated waste heat is almost negligible with or without using CHP.

For GaAs with an 11.04% average efficiency, the extent of the benefits strongly depends on the demands which need to be covered (see Table 7). When CHP is not used, i.e., ‘off’, the waste heat from the fuel cell is not transferred to the buffer tank. For electricity only demand, there is no heat demand so no CHP. In cases when CHP mode is off, it can be seen that natural gas usage is higher. This increases the CO_2_ emission by ~6% at EH and roughly 1% at EHSH.

**Figure 8:**
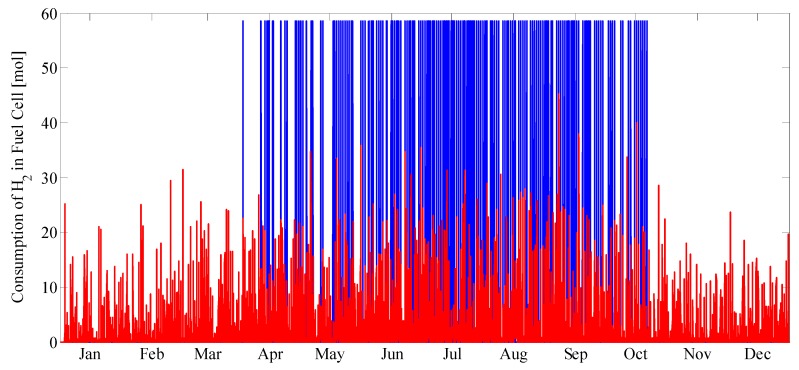
Full (Blue) and partial power mode (Red) of the fuel cell. Full operation has fix quantity hydrogen consumption.

**Figure 9:**
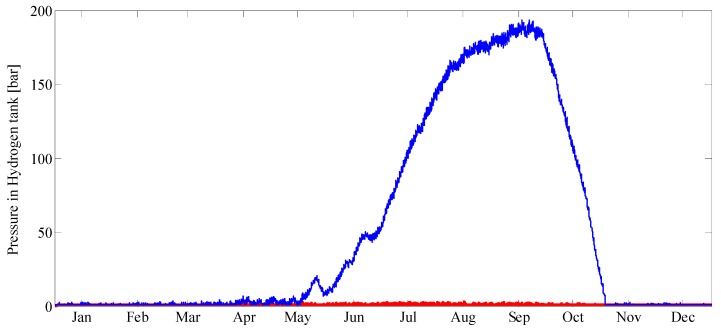
Pressure in the hydrogen storage tank. Blue curve is at 50 m^2^ PEC size while red is only 18 m^2^.

Figure 8 and Figure 9 describe the year-long dynamics of the system. Figure 8 shows the fuel cell operation in the case of full and partial power mode. It can be seen that the partial operation mode has continuous operation each season, while full mode mainly works in the summer months when there is much less space heating requirement.

In Figure 9, the occupancy of the hydrogen storage tank is shown during the four seasons at a PEC size of 18 m^2^ and 50 m^2^ with partial power operation mode of fuel cell and with complete loads (EHSH). The size of the tank depends on the balance of the generation of hydrogen and the consumption. Overall optimisation will of course also require consideration of the economics, capital expenditure and energy savings.

## 4. Conclusions

A model of a photo-electrochemically generated hydrogen for a dwelling CHP system has been presented. Three mathematical approximations of the hydrogen generation system were studied to obtain a view of their suitability for incorporation into the complete system. Case studies were carried out with four electrode materials to analyse energy provision for a household with a full grid connection in the UK environment during a full year. Taking into consideration recent building regulations in the UK [1], a fuel cell-based micro-CHP system was used to cover electricity and heat demands. Results show that, regardless of the efficiency of the electrode types, the generated hydrogen is only at best partially able to cover the overall demands in the case of a representative single band-gap photo-electrode. The techno-economic analysis of the system has focussed mainly on the technical benefits and also on the environmental benefits in terms of reduction in CO_2_ emissions. Due to the lack of commercial products, it is difficult to estimate cost-benefit and payback periods at this time. However, by increasing the efficiency of the PEC unit; for example, with multiband-gap photo-electrode layers, the technology might have a chance to become cost-effective. Alternatively, a PEC unit could be used as a combined solar thermal collector to make better use of the overall solar spectrum. This would increase the overall system efficiency and reduce the hydrogen requirements for thermal demands. Such a combined system could be perceived as a new combined heat and power system. The CO_2_ emission capability is strongly dependent on the sizes of the units and the nature of the demands. In the case of single demand types with low energy band-gap materials, the reduction can be significant. For example, with TiO_2_ the reduction is only 4.3% when there is only electricity demand and 3.1% when the demand is only hot water. The results are 88.9% and 75.6%, respectively, and GaAs electrode material.

## Figures and Tables

**Table 1:** Building loads and solar parameters and settings.

Parameter	Value
**House parameters**:	
Floor area (m^2^):	80
Roof area (m^2^):	80
Openings (m^2^):	20
Walls area (m^2^):	88
Floor thermal transmittance (W m^−2^ °C^−1^):	1.86
Roof thermal transmittance (W m^−2^ °C^−1^):	0.46
Openings thermal transmittance (W m^−2^ °C^−1^):	5.23
Walls thermal transmittance (W m^−2^ °C^−1^):	0.7
Volume of the house (m^3^):	240
Air rate (ACH):	1
Temperature water main (°C):	10
Total heat loss coefficient (W °C^−1^)	431.8
**Location:**	
Latitude (°):	51.53
Longitude (°):	0
**Panel orientation:**	
Tilt angle (°):	51 (South)
**Loads:**	
Electricity (kWh year^−1^):	2540
Space heating (kWh year^−1^):	9610
Volume of hot water (l person^−1^ day^−1^):	68
Hot water (kWh year^−1^)	4326
Space heating set point (°C):	20

**Table 2:** Parameters and settings for the PEC group.

Parameter	Value
η_optical_ (%):	70
LHV of H_2_ (kWh kg^−1^):	33.3
**Electrode:**	**TiO_2_**
λ_g_ (nm):	400
n_D_ (-):	2.496
η_QE_ (%):	58
**Electrode:**	**CdS**
λ_g_ (nm):	517
n_D_ (-):	2.529
η_QE_ (%):	87
**Electrode:**	**GaInP_2_**
λ_g_ (nm):	689
n_D_ (-):	3.15
η_QE_ (%):	66
**Electrode:**	**GaAs**
λ_g_ (nm):	870
n_D_ (-):	3.8
η_QE_ (%):	90
**Compressor:**	
p_max_ (bar):	200
Polytropic coefficient (-):	1.4
η_comp_ (%):	80
**Hydrogen gas tank:**	
p_max_ (bar):	200
Volume of the tank (m^3^):	2

**Table 3:** CHP group device parameters and settings.

Parameter	Value
Hot water tank:	
Volume (litres):	100
Outlet temperature (°C):	60
Buffer tank:	
Temperature max (°C):	80
Volume (litres):	50
Boiler:	
HHV of H_2_ (kJ kg^−1^):	141,900
HHV of Natural Gas (kJ kg^−1^):	54,000
Burning efficiency (%):	90

**Table 4:** Fuel cell parameters and settings.

Parameter	Value
Operation temperature (°C):	80
Operation pressure (atm):	1
Inlet gas compounds in anode (%):	90% H_2_ + 10% H_2_O
Inlet gas compounds in cathode (%):	21% O_2_ + 79% N_2_
Transfer coefficient of H_2_ (-):	0.5
Transfer coefficient of O_2_ (-):	0.3
Exchange current density of H_2_ (A cm^−2^):	0.1
Exchange current density of O_2_ (A cm^−2^):	10^−4^
Internal resistance (Ω cm^2^):	0.019
Limiting current density (A cm^−2^):	2
Mass transfer voltage drop (V):	0.1
Utilization factor	1
Nominal power of fuel cell (kW):	2
Inverter efficiency (%):	90
Nominal voltage (V):	0.7

**Table 5:** Parameters and settings variation for finding the best selection.

Fuel Cell Mode	Buffer Size [litre]	PEC Size [m^2^]	H_2_ Gen [kg]	H_2_ Cons by FC [kg]	H_2_ Cons Boiler [kg]	NG Boiler [kg]	Fuel Cell *Q_gen_* [kWh]	Grid [kWh]	CO_2_ Gen [kg]	*T_max_*/*T_ave_* Buffer [°C]
Full	50	18	93	25	68	838	247	2492	3187	41/13
Full	100	18	93	25	68	837	254	2492	3184	32/13
Full	50	50	257	162	95	688	1610	2200	1705	67/21
Full	100	50	257	162	95	682	1642	2199	1690	63/21
Part	50	18	93	34	59	866	195	1657	3016	27/13
Part	100	18	93	34	59	866	196	1658	3016	23/13
Part	50	50	257	69	188	507	423	795	1675	31/16
Part	100	50	257	69	188	507	428	796	1675	29/16

Notation: gen = generation, cons = consumption, H_2_ = hydrogen, NG = natural gas, max = maximum, ave = average of yearly simulation data.

**Table 6:** System analysis with TiO_2._

Demands	CHP	H_2_ Gen [kg]	H_2_ Cons by FC [kg]	H_2_ Cons Boiler [kg]	H_2_ Rest In Tank [kg]	NG Boiler [kg]	Fuel Cell *Q_gen_* [kWh]	Grid [kWh]	CO_2_ Gen [kg]	*T_max_*/*T_ave_* Buffer [°C]
E	-	4	3.8	0	0.2	0	0	2427	1273	10/10
H	-	4	0	3.8	0.2	312	0	0	772	10/10
EH	On	4	3.7	0.1	0.2	321	7.4	2430	2070	10/10
EH	Off	4	3.7	0.1	0.2	321	0	2430	2072	10/10
EHSH	On	4	3.4	0.4	0.2	1032	6.5	2438	3837	10/10
EHSH	Off	4	3.4	0.4	0.2	1032	0	2438	3838	10/10

Notation: E = only electricity, H = only hot water, EH = electricity and hot water, EHSH = electricity, hot water and space heating.

**Table 7:** System analysis with GaAs.

Demands	CHP	H_2_ Gen [kg]	H_2_ Cons by FC [kg]	H_2_ Cons Boiler [kg]	H_2_ Rest in Tank [kg]	NG Boiler [kg]	Fuel Cell *Q_gen_* [kWh]	Grid [kWh]	CO_2_ Gen [kg]	*T_max_*/*T_ave_* Buffer [°C]
E	-	93	89.6	0	3.4	0	0	282	148	10/10
H	-	93	0	92.6	0.4	78	0	0	195	10/10
EH	On	93	50.9	41.7	0.4	190	309	1239	1121	31/15
EH	Off	93	49.7	42.9	0.4	209	0	1270	1184	10/10
EHSH	On	93	34	58.5	0.5	866	195	1657	3016	27/13
EHSH	Off	93	34	58.7	0.3	879	0	1664	3052	10/10

Notation: E = only electricity, H = only hot water, EH = electricity and hot water, EHSH = electricity, hot water and space heating.
